# Widely Tunable Narrow-Linewidth Continuous-Wave MIR Laser at 2.2–5.1 μm

**DOI:** 10.3390/ma19143055

**Published:** 2026-07-16

**Authors:** Shuai Ye, Feifei Wang, Yongping Yao, Runze Liang, Hongkun Nie, Zhiyuan Zuo, Baitao Zhang

**Affiliations:** 1 State Key Laboratory of Chips and Systems for Advanced Light Field Display, Center for Optics Research and Engineering, Shandong University, Qingdao 266237, China; 2 Shandong Provincial Key Laboratory of Laser Technology and Application, Shandong University, Qindao 266237, China; 3 Shandong Best-Ray Laser Technology Co., Ltd., Jinan 250199, China; 4 State Key Laboratory of Crystal Materials, Institute of Novel Semiconductors, Shandong University, Jinan 250100, China

**Keywords:** mid-infrared lasers, widely tunable, narrow-linewidth

## Abstract

A widely tunable narrow-linewidth continuous-wave (CW) mid-infrared (MIR) laser was realized with a PPMgO:LN-based optical parametric oscillator (OPO) pumped by a 1060 nm single-frequency fiber laser. The MIR laser tuning with a range from 2250.7 to 5092.7 nm was obtained using the two multi-period PPMgO:LN crystals. The output power was larger than 520 mW@2250.7–4000 nm, 160 mW@4000–4300 nm, and 15 mW@4300–5095.4 nm, where the maximum output power of 4.64 W@2500 nm was obtained with an optical-to-optical conversion efficiency of 15.5%. The linewidth was measured to be ~0.41 MHz at 3300 nm using the delayed self-homodyne method. The output power stability was measured to be RMS = 1.43% for 8 h. The beam quality factors at 2786, 3360, and 4453 nm were 1.18, 1.19, and 1.26, measured using the knife-edge method. Finally, an engineering prototype was developed using high-reliability engineering design and system integration. Our results will play a significant role in the development of widely tunable narrow-linewidth MIR lasers.

## 1. Introduction

Locating at the “gas fingerprint area” and “atmospheric window region”, mid-infrared (MIR) lasers operating at the wavelength band ranging from 2 to 5 μm have attracted much attention and have been widely used in fields such as environmental monitoring, medical diagnosis, materials processing, military and frontier science, etc. [1,2,3,4]. In particular, narrow-linewidth 2–5 μm continuous-wave (CW) MIR lasers play a significant role in applications such as gas monitoring/detection, precision spectroscopy, polymer cutting and welding, and so on [5,6,7,8].

Compared with other techniques such as quantum cascade lasers, ion-doped crystal-based direct-emitting solid-state lasers, optical super-lattice-based optical parametric oscillators (OPOs) have been proven to be the most efficient and commonly used technique for generating widely tunable CW 2–5 μm MIR laser output, having the advantages of high power, high efficiency, a wide tuning range, low power consumption, easy integration, robustess, etc. [9,10,11,12]. In 2006, Henderson et al. used a 1083 nm single-frequency fiber laser to pump a MgO:PPLN OPO-generating single-frequency CW laser output in the 2.65–3.2 µm band with a linewidth of ~1 MHz under an output power of 500 mW. The absorption spectra of CH_4_ and CO_2_ gases were measured using this light source [13]. In 2008, Vainio et al. used a four-mirror cavity structure to achieve Watt-level single-frequency CW MIR laser output in the range of 2.7–3.45 µm without mode-hopping for several hours [14]. S.T. Lin et al. used a single-resonant four-mirror cavity structure to achieve single-frequency CW MgO:PPLN OPO with a linewidth of 5 MHz by inserting a standard cell into the cavity, and they observed bistable phenomena [15]. In 2011, Ebrahim-Zadeh et al. realized CW single-frequency OPO operation in the range of 3.032–3.462 μm based on PPSLT, with an output power greater than 2 W [16]. In 2013, Raybaut et al. used nested double-cavity dual-resonant MgO:PPLN OPO to obtain a single-frequency 3.3–3.5 μm parametric light output, which was used for trace gas detection [17]. In 2014, Zeil et al. used a ring cavity OPO combined with the frequency selection effect of a volume Bragg grating to achieve single-frequency 3.4 μm laser output with a power of 11 W [18]. In 2015, Ricciardi et al. used an active servo system to obtain a continuous-wave single-frequency 2.7–4.2 μm output with a linewidth less than 1 kHz in a ring cavity PPLN OPO [19]. In 2015, Wei Xingbin et al. used a volume Bragg grating (VBG) to compress the linewidth and obtained 51.7 W narrow-linewidth 2907 nm parametric light [20].

In 2021, our group used a four-mirror ring cavity double-resonant MgO:PPLN OPO to achieve a wide-tuning and narrow-linewidth 2.2–5.1 μm MIR laser output [11]. The pump source was a home-made high-power 1064.2 nm all-solid-state single-frequency laser in which a non-planar ring oscillator was selected as the seed and a dual-end pumped Nd:YVO_4_ structure was designed for the pre-amplifier while a direct-pumped densely folded Nd:YVO_4_ Innoslab was used for the master amplifier [21]. Therefore, the pump laser structure was very complex. By contrast, single-frequency fiber lasers have advantages of high beam quality, high power output, high efficiency, compact size, low maintenance, and stable long-term operation, being the most suitable pump source for narrow-linewidth PPLN OPO. A comprehensive quantitative comparison is listed in Table 1.

In this paper, by using a single-frequency CW fiber laser to pump multi-period MgO:PPLN, combined with tuning methods such as crystal period and temperature tuning and standard cell tuning and PZT tuning, wide-tuning and narrow-linewidth mid-infrared laser output in the range of 2250.7 to 5092.7 nm was achieved. The maximum output power of 4.64 W@2500 nm was obtained with an optical-to-optical conversion efficiency of 15.5%. The linewidth was measured to be ~0.41 MHz at 3300 nm, and the beam quality factors at 2786, 3360, and 4453 nm were 1.18, 1.19, and 1.26. Moreover, an engineering prototype was also developed.

## 2. Materials and Methods

**Figure 1:**
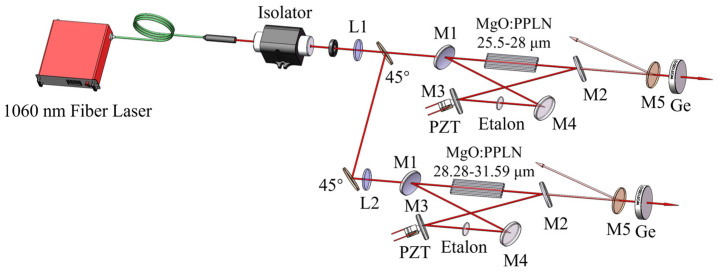
Experimental setup of the widely tunable narrow-linewidth dual-channel MgO:PPLN OPO.

The experimental setup is shown in Figure 1. The OPO pump source has a central wavelength of 1060 nm, an output power of 32.3 W, a beam quality factor of M^2^ < 1.2, and a fiber laser output linewidth of 50 kHz. To achieve wide-tuning output in the range of 2–5 μm, a dual-channel OPO was used and switched by a 45° mirror coated with a high-reflection coating at 1064 nm. The pump laser was shaped by two lenses with focal lengths of 175 and 60 mm into the PPLN crystal with a focused spot radius of about 70 μm. Two multi-period MgO:PPLN with MgO doping concentrations of 5 mol%, dimensions of 50 × 7.4 × 1 mm^3^ and 50 × 10 × 1 mm^3^, and polarization periods of 25.5–28 μm and 28.28–31.59 μm were used. The two end faces of the crystals were antireflection (AR) coated. The superlattice crystals were placed in a self-made high-precision temperature control device (tuning accuracy ±0.01 °C) with a temperature tuning range of 40–200 °C. The temperature control furnace was placed on a high-precision stepper motor for fine switching of the crystal period. To facilitate the insertion of frequency selection devices in the cavity and improve OPO stability, a single-resonant four-mirror ring cavity structure was used. The four cavity mirrors were composed of two concave mirrors, M1 and M2 (r = 100 mm), and two plane mirrors, M3 and M4. The two sets of cavity mirrors were coated with high-reflection (HR) coatings at 1.3–1.45 μm, high-transmission (HT) coatings at 4.1–5.1 μm, and HR coatings at 1.45–2.0 μm and HT coatings at 2.3–4.15 μm, ensuring single-frequency operation of the signal light in the laser cavity. The total cavity length was 42 cm, and the angle between the cavity mirrors and the optical path was about 15°. The cavity was designed to have a waist radius of 69 μm for the resonant signal beam, satisfying the optimal focusing condition. In each of the two OPO channels, a 400 μm thick uncoated YAG standard cell was inserted at the second waist between M3 and M4, which acted as a frequency selector and improved the long-term stability of the OPO by providing a free spectral range (FSR~206 GHz). In addition, a PZT was added to achieve fine wavelength tuning of the OPO. The pump light and the output idler light were separated using a beamsplitter M5, and a germanium plate was added to further filter out unnecessary visible light.

**Figure 2:**
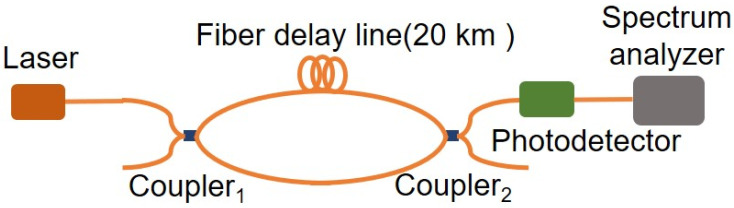
Schematic diagram of the delayed self-homodyne setup for spectral linewidth measurement.

To characterize the spectral linewidth at 3300 nm idler output, we set the MgO:PPLN crystal period to 30.49 µm and crystal temperature to 107 °C, generating signal light at 1570 nm and target idler light at 3300 nm. The resonant signal light was coupled into a fused silica single-mode fiber and split into two branches via a fiber coupler. One branch passed through a 20 km long-delay fiber as the reference beam, while the other branch served as the test beam without extra delay. The two beams were recombined by another fiber coupler to generate a beat-frequency interference signal. A high-speed InGaAs photodetector (PDA05CF2, Thorlabs, Newton, NJ, USA) converted the optical beat signal into an electrical signal, which was collected and analyzed using a frequency analyzer (N9000A, Agilent Technologies, Santa Clara, CA, USA). The measured spectral data was fitted with a Lorentzian function, and the full width at half maximum (FWHM) of the fitted curve corresponds to the laser’s single-frequency spectral linewidth. Figure 2 shows the experimental setup.

## 3. Results

**Figure 3:**
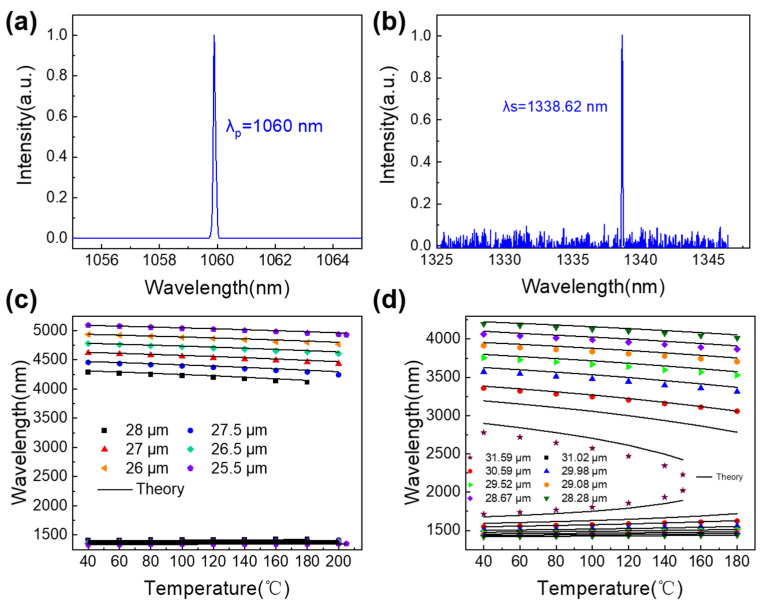
PPLN-OPO wavelength tuning range: (**a**) Pump source spectrum. (**b**) Signal spectrum at the 25.5 μm period, 40 °C. (**c**) Wavelength tuning range of the 25.5–28 μm period. (**d**) Wavelength tuning range of the 28.28–31.59 μm period.

The wavelength tuning range that the OPO can achieve was explored first. The spectrum of the OPO pump source is shown in Figure 3a. For crystal 1, with a polarization period of Λ = 25.5 μm and a temperature of 40 °C, the OPO could oscillate at a pump power of 27 W. At this point, the oscillating signal wavelength was measured to be 1338.62 nm behind the M3 cavity mirror using a spectrometer, as shown in Figure 3b. According to energy conservation, the corresponding maximum idler wavelength that the device can achieve was calculated to be 5092.73 nm. Furthermore, to investigate the continuous wavelength tunability of the OPO, the period and temperature of the 14-period MgO:PPLN crystals with polarization periods of 25.5–28 μm and 28.28–31.59 μm were controlled, achieving continuous tunable mid-infrared laser output of the idler laser ranging from 2250.7 to 5092.7 nm with a tuning range of 2842 nm. The wavelength tuning curves of the two OPO channels are shown in Figure 3c and Figure 3d, respectively, where the solid line represents the theoretical calculation results obtained from the corresponding Sellmeier equation, which agrees well with the experimental measurements. The optical spectrum analyzer (OSA 207C, Thorlabs, Newton, NJ, USA) and power meter (S440C, Thorlabs, Newton, NJ, USA) were calibrated before each group of experiments according to factory standard calibration procedures; wavelength calibration error was less than ±0.5 nm, and power measurement error was less than ±2%.

**Figure 4:**
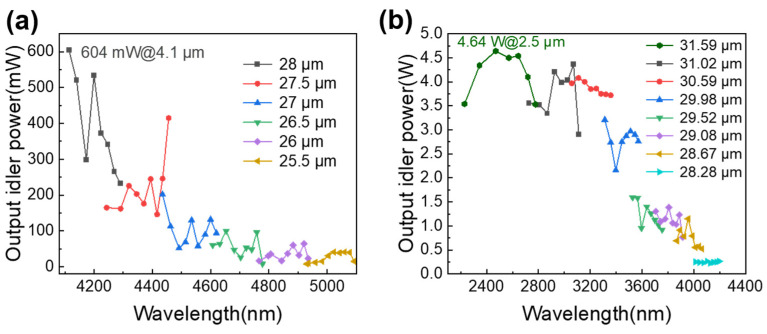
Idler output power at different wavelengths: (**a**) OPO channel 1, 4.1–5.1 μm; (**b**) OPO channel 2, 2.2–4.1 μm.

Figure 4 shows the idler laser output power curves corresponding to different wavelengths within the corresponding wavelength tuning range of the two OPOs. In order to prevent damage to MgO:PPLN, the MgO:PPLN crystal period and temperature were adjusted at a low pump power level. From Figure 3a, it can be seen that the OPO output power is greater than 160 mW within the wavelength range of 4.1–4.3 μm and greater than 15 mW in the range of 4.3–5.1 μm. Among them, the maximum output power was obtained at 4.1 μm with a period of 28 μm and a temperature of 180 °C. From Figure 3b, it can be seen that, within the tuning range of 2.2–4.2 μm, over 60% of the idler laser output power is greater than 1 W, and the output power within the range of 2.2–4.0 μm is greater than 520 mW. Among them, at a crystal cycle of 31.59 μm and a temperature of 120 °C, the maximum output power of 4.64 W is obtained at an idler laser wavelength of 2500 nm, and the corresponding optical-to-optical conversion efficiency is 15.5%.

**Figure 5:**
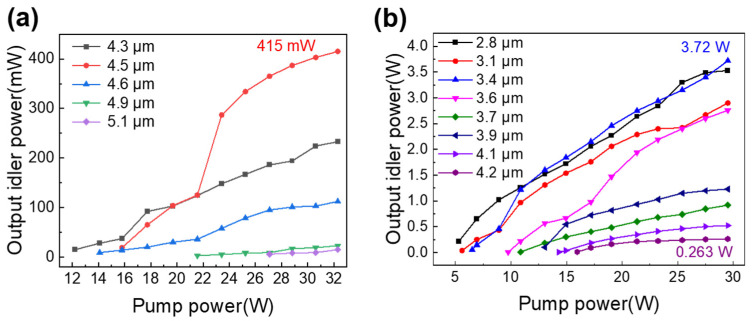
Special wavelength output power: (**a**) OPO channel 1, 4.1–5.1 μm; (**b**) OPO channel 2, 2.2–4.1 μm.

**Figure 6:**
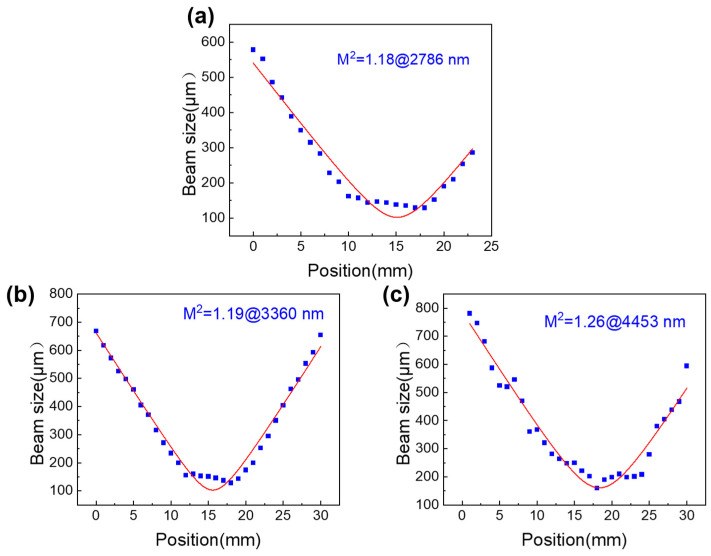
Beam quality measurement: (**a**) 2786 nm; (**b**) 3360 nm; (**c**) 4453 nm.

Figure 5 shows the special wavelength output power. For OPO channel 1, due to the increasing absorption of the PPLN crystal above 4 μm, the oscillation threshold of the OPO is generally high. At 5 μm, the oscillation threshold reaches 27 W. At maximum pumping power, the maximum and minimum output of the parametric light are obtained at 4.5 μm and 5.1 μm, respectively. The corresponding output power and optical-to-optical conversion efficiency are 415 mW/15 mW and 1.3%/0.046%. For OPO channel 2, as the output wavelength increases from 2.8 μm to 4.2 μm, the oscillation threshold of the OPO increases from 5.3 W to 15.9 W. At an input power of 30 W, the maximum output power is obtained at 3.4 μm, reaching 3.72 W, with a corresponding optical-to-optical conversion efficiency of 12.4%. The minimum output power of 263 mW is obtained at 4.2 μm, with a corresponding optical-to-optical conversion efficiency of 0.9%. The output power instability is measured to be RMS = 1.43% at 2.7 μm for 8 h operation. The excellent power stability of the OPO is due to the design of the four-mirror ring cavity and the insertion of a standard sample. Beam quality measurements were carried out at 2786, 3360, and 4453 nm with the beam quality factor M^2^ of 1.18, 1.19, and 1.26, respectively, as shown in Figure 6.

In addition, the effect of YAG etalon insertion on the OPO was investigated. The OPO oscillation signal spectrum was recorded every 5 s using a spectrometer. Before the insertion of the standard sample, the OPO showed clear multimode oscillation, while no significant mode hopping was observed within 20 s after the insertion of the standard sample. This indicates that the insertion of the standard sample can effectively perform longitudinal mode selection and improve the long-term stability of the OPO wavelength. The experiment also showed that, by changing the insertion angle of the standard sample within a certain range of ±10°, the oscillation wavelength can be finely tuned. When the insertion angle of the standard sample is too large, the insertion loss increases significantly, leading to a significant decrease in the output power of the OPO.

**Figure 7:**
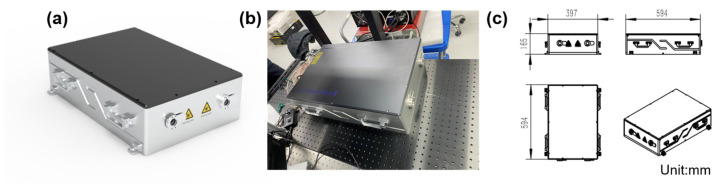
A 2–5 μm wide tuned narrow-linewidth OPO laser: (**a**) rendering, (**b**) physical object, and (**c**) engineering drawing.

A delayed self-homodyne method was employed to measure the spectral bandwidth of the OPO output [21,27]. The intrinsic instrumental linewidth limit of this test system is ~50 kHz, far below the measured laser linewidth of ~0.41 MHz, which verifies the validity of our linewidth result. Moreover, an engineering prototype was developed using high-reliability engineering design and system integration. The engineering design of the widely tunable MIR laser is shown in Figure 7 with dimensions of 600 mm × 390 mm × 165 mm.

## 4. Conclusions

In summary, we report a compact single-frequency fiber laser pumped continuous-wave MgO:PPLN OPO featuring a wide tuning range, narrow spectral linewidth, and near-diffraction-limited beam quality. The system achieves a continuous idler tuning band spanning 2.25–5.09 μm, delivering a peak output power of 4.64 W at 2500 nm with an overall optical-to-optical conversion efficiency of 15.5%. The measured spectral linewidth of the 3300 nm idler output is ~0.41 MHz via the delayed self-homodyne method. The beam quality factors M^2^ are characterized as 1.18 at 2786 nm, 1.19 at 3360 nm, and 1.26 at 4453 nm, confirming excellent Gaussian beam performance across most of the operating band. An integrated engineering prototype with overall dimensions of 600 mm × 390 mm is fabricated for practical deployment. Nevertheless, this setup still suffers from inherent limitations: quantitative beam-pointing drift cannot be measured due to the lack of dedicated test instruments in our laboratory, and the idler power degrades drastically above 4.3 μm because of strong MgO:PPLN mid-infrared absorption. In future work, we will introduce beam-drift testing equipment and optimize crystal thermal management to further stabilize long-term output. This work provides a feasible miniaturized technical route for high-performance mid-infrared coherent sources, and holds great application prospects in high-precision trace gas detection, spectroscopic analytical chemistry, and infrared photoelectric sensing.

## Figures and Tables

**Table 1:** Summary of CW MIR OPOs based on PPLN.

Num.	Tuning Range	Output Power	Linewidth	Beam Quality	Stability	Ref.
1	2620–4160 nm	2.11 W@3170 nm	1.8 nm	-	<5%@10 min	[22]
2	2500–3600 nm	64 mW@3500 nm	-	-	<3%@1 h	[23]
3	3100–4450 nm	250 mW@3365 nm	~0.2 nm	M^2^ < 1.3	<±3%@30 min	[24]
4	3833–4114 nm	187 mW@3833.2 nm	6.4 nm	M^2^ ≈ 1.3	1.5%@2 h	[25]
5	2890–3574 nm	4.06 W@3014 nm	<665 kHz	M_x_^2^ = 1.25 M_y_^2^ = 1.33	0.8%@30 min	[26]
6	2250–5092 nm	4.64 W@2500 nm	~410 kHz	M^2^ < 1.3	1.43%@8 h	This work

## Data Availability

The original contributions presented in this study are included in the article. Further inquiries can be directed to the corresponding author.
